# Inhibitory Effect of Essential Oils on *Aspergillus ochraceus* Growth and Ochratoxin A Production

**DOI:** 10.1371/journal.pone.0108285

**Published:** 2014-09-25

**Authors:** Huijuan Hua, Fuguo Xing, Jonathan Nimal Selvaraj, Yan Wang, Yueju Zhao, Lu Zhou, Xiao Liu, Yang Liu

**Affiliations:** Institute of Agro-Products Processing Science and Technology, Chinese Academy of Agricultural Sciences/Key Laboratory of Agro-Products Processing, Ministry of Agriculture, Beijing, P. R. China; University of Nebraska-Lincoln, United States of America

## Abstract

Ochratoxin A (OTA) is a mycotoxin which is a common contaminant in grains during storage. *Aspergillus ochraceus* is the most common producer of OTA. Essential oils play a crucial role as a biocontrol in the reduction of fungal contamination. Essential oils namely natural cinnamaldehyde, cinnamon oil, synthetic cinnamaldehyde, *Litsea citrate* oil, citral, eugenol, peppermint, eucalyptus, anise and camphor oils, were tested for their efficacy against *A. ochraceus* growth and OTA production by fumigation and contact assays. Natural cinnamaldehyde proved to be the most effective against *A. ochraceus* when compared to other oils. Complete fungal growth inhibition was obtained at 150–250 µL/L with fumigation and 250–500 µL/L with contact assays for cinnamon oil, natural and synthetic cinnamaldehyde, *L. citrate* oil and citral. Essential oils had an impact on the ergosterol biosynthesis and OTA production. Complete inhibition of ergosterol biosynthesis was observed at ≥100 µg/mL of natural cinnamaldehyde and at 200 µg/mL of citral, but total inhibition was not observed at 200 µg/mL of eugenol. But, citral and eugenol could inhibit the OTA production at ≥75 µg/mL and ≥150 µg/mL respectively, while natural cinnamaldehyde couldn’t fully inhibit OTA production at ≤200 µg/mL. The inhibition of OTA by natural cinnamaldehyde is mainly due to the reduction in fungal biomass. However, citral and eugenol could significant inhibit the OTA biosynthetic pathway. Also, we observed that cinnamaldehyde was converted to cinnamic alcohol by *A. ochraceus*, suggesting that the antimicrobial activity of cinnamaldehyde was mainly attributed to its carbonyl aldehyde group. The study concludes that natural cinnamaldehyde, citral and eugenol could be potential biocontrol agents against OTA contamination in storage grains.

## Introduction

*Aspergillus* species are frequent contaminants of low-moisture foods (water activity <0.75). Increase in metabolic activity of *Aspergillus* species causes the food to spoil leading to enormous economic loss. Besides, some *Aspergillus* species can produce toxic secondary metabolites: like aflatoxins, ochratoxins and they affect the food safety. Ochratoxins are nephrotoxic, potentially carcinogenic mycotoxins in nature [1]–[3]. Ochratoxin is common contaminant in food products such as cereals, coffee, wine, beer, and spices. At least 20 different ochratoxin analogs have been detected in the recent years. Ochratoxin A (OTA) is the commonly produced analogue and highly toxic [4]. OTA is mainly produced by *Aspergillus ochraceus*, *Aspergillus carbonarius*, *Aspergillus westerdijkiae*, *Aspergillus niger*, *Penicillium nordicum* and *Penicillium verrucosum* [5]. *A. ochraceus* is responsible for OTA contamination in rice, wheat, oats, coffee, and beverage. *A. carbonarius* and *A. niger* are responsible for OTA in grapes, raisins, and wine [6], [7].

As the most toxic and prevalent toxin of the ochratoxins group, OTA is highly neurotoxic, teratogenic, embryotoxic and genotoxic, immunosuppressive and immunotoxic in nature [8], [9]. OTA was rated as potential carcinogen (group 2B) by the International Agency for Research on Cancer (IARC, 1993). European Union proposed a maximum tolerable level of 5 µg/kg OTA in cereal grains (European Commission, 2006), and it is similar to China.

Several strategies have been applied to prevent and control the growth of OTA producing fungi in grains. Chemical based control remains the common measure to reduce the incidence of post-harvest contamination in various foods. Antifungal chemicals like benzimidazoles, aromatic hydrocarbons, and sterol biosynthesis inhibitors are often used. However, the application of them increases the risk of toxic residues in foods [10]–[14]. Furthermore, the use of chemical fungicides often leads to fungal resistance [15]. Therefore, a huge effort is made in the recent years to limit the use of fungicides in grains and foods. Natural antimicrobial or antifungal substances are promising to replace these synthetic fungicides.

Essential oils (EOs) from plants have a broad spectrum of antifungal activity [16]–[20]. Bluma & Etcheverry (2008) [21] screened 41 aqueous and ethanolic extracts and 22 different EOs for their antifungal effects against *Aspergillus* section *Flavi* strains and concluded that boldo, poleo, clove, anise and thyme oils could be a potential antifungal agent. In their study, EOs screened for antifungal effect was by adding them to media followed by diffusion. Boldo, poleo and clove oils could inhibit the growth of *A. niger* and *A. carbonarius* by affecting the OTA biosynthesis pathway [22]. Smaller compounds such as monoterpenes were found to highly effective when used as headspace volatiles [23]. Vapor exposure to these oils could be an effective, non-toxic biopreservatives against OTA contamination in stored grains and foods.

The objectives of our study were to: (a) examine the efficacy of different EOs like cinnamon oil, natural cinnamaldehyde, synthetic cinnamaldehyde, *L. citrate* oil, citral, eugenol, eucalyptus, anise, peppermint and camphor oils against *A. ochraceus* and OTA accumulation on malt extract agar (MEA) and in yeast extract sucrose (YES) broth; (b) Using contact and headspace volatile assays to analyze their antifungal effect; and (c) cinnamaldehyde transformations in YES broth with *A. ochraceus*.

## Materials and Methods

### Essential oils

Ten EOs were used in the present study: cinnamon oil (85% cinnamaldehyde), natural cinnamaldehyde (95%), synthetic cinnamaldehyde (99%), *Litsea citrate* oil (85% citral), citral (96%), eugenol (99% eugenol), eucalyptus (80% cineole), anise (92% anethole), peppermint (50% menthol), and camphor (55% bomeol) oils were purchased from Jiangxi Xue Song Natural Medicinal Oil Co., Ltd, P. R. China.

### Fungal strain and culture conditions

*A. ochraceus* 3.4412 producing OTA was gifted by Prof. Kunlun Huang (College of Food Science and Nutritional Engineering, China Agricultural University, Beijing, P. R. China) and maintained in potato dextrose agar (PDA) for 2 weeks and stored at 4°C. The strain has previously shown to produce 1648.3 ng/g and 1536.1 ng/mL of OTA in MEA plates and YES broth, respectively.

### Effect of the essential oils on *A. ochraceus* growth with fumigation

Evaluation of the antifungal activity of the EOs by fumigation was performed according to Soliman and Badeaa (2002) [24] with minor modifications. Briefly, 50 µL of 10^5^ conidia/mL suspended in 0.01% Tween 20 was plated on 20 mL of MEA in a plastic Petri plate. EOs at different concentrations (25–1500 µL/L) was added to 10 mm sterile filter disks placed on cover of the Petri plate. The Petri plate was sealed by using Parafilm and incubated for 20 days at 28±1°C (five replicates for each treatment). The colonies were measured every 2 days in two directions at right angles to each other to obtain the mean diameter. Blanks were used in triplicates.

After the incubation period, the plates showing no growth were incubated for a further 7 days under the similar temperature conditions followed by replacing new filter disk without EOs. If *A. ochraceus* growth is observed, there is a fungistatic effect, whereas if no growth occurs, the effect is fungicidal.

### Effect of the essential oils on *A. ochraceus* growth by contact assay

The effect of EOs on *A. ochraceus* growth on MEA was studied according to Passone et al. (2012) [23] with minor modifications. MEA plates containing 20–1500 µL/L of EO was prepared by adding EO to 20 mL autoclaved medium. 50 µL of 10^5^ conidia/mL suspended in 0.01% Tween 20 was spotted on the center of each plate. Plates were sealed using Parafilm and incubated for 20 days at 28±1°C with five replicates for each treatment. Two measurements were made at right angles to each other for each colony every 2 days to obtain mean diameter. Cultures from plates showing no growth were transferred to plates containing medium without EO to determine fungistatic and fungicidal effects. Blanks were used in triplicates.

### Determination of the minimum inhibitory concentration by contact assay

The minimum inhibitory concentration (MIC) of EOs was determined by the broth dilution method according to Yamamoto-Ribeiro et al. (2013) [20] with minor modifications. The EOs were diluted in 0.001% Tween-80 and tested at final concentrations of 40−40,000 µL/L. For each essential oil, 500 µL of 4×10^5^ spores/mL of *A. ochraceus* conidia was added in RPMI-1640 medium (0.5 mL). Tubes were incubated at 35°C for 72 h with agitation. The lowest concentration showing no visible growth was designated as MIC. Cells from the tubes showing no growth were subcultured on PDA plates to determine if the inhibition was reversible or permanent. Positive controls were performed in medium with only the cell suspension.

### Scanning electron microscopy (SEM)

SEM was performed according to a modified method from Xing et al. (2014) [25]. *A. ochraceus* was treated with natural cinnamaldehyde (100 µL/L), citral (100 µL/L) and eugenol (250 µL/L) on MEA medium with fumigation, respectively, Then the treated *A. ochraceus* mycelia were collected, and the fungi were mixed with formaldehyde, washed with PBS buffer, and dehydrated with gradient ethanol solutions (30, 60, 80, 90 and 100%, keeping the mycelia for a longer duration in 100%). The samples were subjected to critical point drying, subsequently, the fungal mycelia were prepared and observed under Hitachi S-750 SEM.

### Effect of essential oils on ergosterol biosynthesis and ochratoxin A production

The effect of EOs on ergosterol biosynthesis and OTA production were determined according to a modified method from Kocić-Tanackov et al. (2012) [26]. Fifty milliliters of sterile YES broth (yeast extract 20 g, sucrose 150 g, MgSO_4_·7H_2_O 0.5 g, distilled water 885 mL) were poured in flask (150 mL). Natural cinnamaldehyde, citral and eugenol were aseptically added to the YES broth to obtain the following concentrations: 0, 25, 50, 75, 100, 150, or 200 µg/mL. Three 5 mm diameter discs of *A. ochraceus* mycelia from PDA medium were inoculated to the YES broth., and incubated at 28°C for 7 days under agitation in a ZWY-2102C incubator (Zhicheng, Shanghai, China). After the incubation, YES broth containing *A. ochraceus* mycelia was filtered through Whatman filter No. 1 and washed with distilled water. Mycelia were used for ergosterol determination and the filters for OTA determination. Five replicates were performed for the experimental and control group.

### Ergosterol and ochratoxin A standards

Ergosterol (Sigma Chemical, St. Louis, MO, USA) solutions (20 µg/mL) were prepared in absolute ethanol PA (Merck, Germany) and 100 µg/mL of OTA (Sigma Chemical, St. Louis, MO, USA) was prepared in acetonitrile:water (50∶50, v/v) and stored in amber vials at −18°C.

### Ergosterol extraction and determination by high-performance liquid chromatography

Ergosterol was extracted according to Silva, Corso, and Matheus (2010) [27] and transferred to a Falcon tube containing 20 mL methanol, 5 mL absolute ethanol PA, and 2 g potassium hydroxide. The solution was stirred for 5 min and placed in a water bath at 70°C for 40 min. After cooling at room temperature, 5 mL of distilled water was added and the solution was centrifuged at 1735×*g* for 12 min. For final extraction, *n*-hexane was added in an equal volume to the supernatant and the organic fraction was collected in an amber glass vial and evaporated with 99.99% nitrogen flow at 60°C. The residue obtained was stored at −18°C.

Ergosterol quantification was according to Yamamoto-Ribeiro et al. (2013) [20] with minor modifications. High performance liquid chromatography (HPLC) was performed with a Water 2695 (Waters Corporation, Milford, MA, USA) with an ultraviolet/visible spectrum Waters 2475 detection system at wavelength of 282 nm. Extract was resuspended in 1 mL of absolute ethanol and 100 µL was injected. For the mobile phase, methanol was used at a flow rate of 1.5 mL/min. Reverse-phase HPLC separation was on a Agilent TC-C18 (Agilent, Santa Clara, USA) with a 5 µm particle size column (250×4.6 mm). Retention time was 4.6 min and detection limit was 0.15 µg/mL.

### Ochratoxin A extraction and determination by high-performance liquid chromatography

OTA was extracted according to Passone et al. (2012) [23] with minor modifications. Cell-free extracts (10 mL mixed with 10 mL methanol) were stirred in shaker tubes for 1 min, centrifuged at 14,000 rpm for 10 min and filtered (Titan 2 HPLC Filter Green; 17 mm and 0.45 µm). The eluate was evaporated to dryness with 99.99% nitrogen flow at 60°C. The residue was redissolved in mobile phase (acetonitrile/water/acetic acid, 99∶99∶2) and the extract was injected into a tube or stored at −18°C.

OTA production was detected and quantified by the methodology proposed by Scudamore and MacDonld (1998) [28] with minor modifications. Reversed phase HPLC with a Waters 2475 fluorescence detection (λexc 330 nm; λem 460 nm) was applied. Agilent TC-C18 column (250×4.6 mm, 5 µm particle size) was used. The mobile phase (acetonitrile/water/acetic acid, 99∶99∶2) was pumped at 1.0 mL/min. Injection volume was 50 µL and retention time was around 6 min. Standard curves were constructed with 5 to 100 ng/g OTA (Sigma Chemical, St. Louis, MO, USA). Toxin was quantified by correlating peak areas of sample extracts and standard curves. Mean recovery was calculated by spiking with MEA at 5 to 100 ng/g and was estimated at 89.2±9.7%. The lowest detection limit was 1 ng/g.

### Cinnamaldehyde determination by high-performance liquid chromatography

The variations in concentrations of cinnamaldehyde and its possible metabolite cinnamic alcohol in YES broth with *A. ochraceus* (treatment) were determined by RP-HPLC analysis. YES broth containing 100 µg/mL of cinnamaldehyde was inoculated with *A. ochraceus* and incubated at 28°C with shaking at 180 rpm. YES broth without fungi inoculation by with cinnamaldehyde was the negative control and YES broth with fungi inoculation by without cinnamaldehyde was the positive control. Samples of 10 mL were collected at 0, 2, 4, 8, 12, 24, 48, 72, and 96 h and centrifuged at 10,000×*g* for 10 min at 4°C. Cell pellets were resuspended in methanol (Fisher) and sonicated for 15 min in a Bransonic bath (5510; Branson, Grass Valley, CA). The suspension was filtered through 0.2 µm nylon syringe filter units (Fisher) and analyzed for HPLC. Supernatant were mixed with 10 mL ethyl acetate (Sigma-Aldrich) to avoid interference by materials of YES broth in HPLC detection. The mixture was mixed on vortex for 3 min. After a 3 min rest, the clear ethyl acetate phase at the top was collected and the procedure was repeated twice. Ethyl acetate extracts from negative controls were not centrifuged. Pooled ethyl acetate extracts from each 10 mL sample were evaporated using a rotary evaporator (Yarong, SY2000; Shanghai, China), and the residue was dissolved in 20 mL methanol. The solution was further diluted in methanol if necessary, filtered through a 0.2 µm nylon syringe filter unit, and immediately used for HPLC analysis. Extracts from duplicate supernatants and corresponding cell pellets were analyzed separately.

Analysis used a Waters 2695 reversed-phase HPLC with a Waters 2487 UV detector at 280 nm and empower system controller. Data were processed using Waters LC-module 1 millennium software, version 32. A reverse-phase Agilent TC-C18 column was used. The mobile phases used for separation were: A, 1% acetic acid in methanol; and B, 1% acetic acid in water. A gradient flow of mobile phases (0 to 10 min at 15% A, 85% B; 10 to 13 min at 50% A, 50% B; 13 to 16 min at 60% A, 40% B; 16 to 24 min at 65% A, 35% B and 24 to 40 min at 15% A, 85% B) at 1 mL/min was used. Retention times and standard curves for cinnamaldehyde and cinnamic alcohol were obtained by analyzing 10 µL filtered standard solution (0.5 to 50 µg/mL) of each compound. Curve linearity and correlation coefficients were calculated from peak areas at each standard concentration. Ten µL extract from treatment samples or positive or negative controls was injected. Methanol extracts of cells were injected separately at the same level with supernatants and final concentrations of cinnamaldehyde or cinnamic alcohol were obtained by combining values for cell extracts and extracts from corresponding supernatants.

### Statistical analyses

All the experiments results were evaluated using analysis of variance (ANOVA) for multiple comparisons followed by the Turkey test. Differences were considered significant at *p*<0.05. The growth of fungal cultures with different concentrations of the EOs was compared with growth of a control with no EO.

## Results

### Inhibitory effect of ten essential oils on *A. ochraceus* growth with fumigation

**Figure 1:**
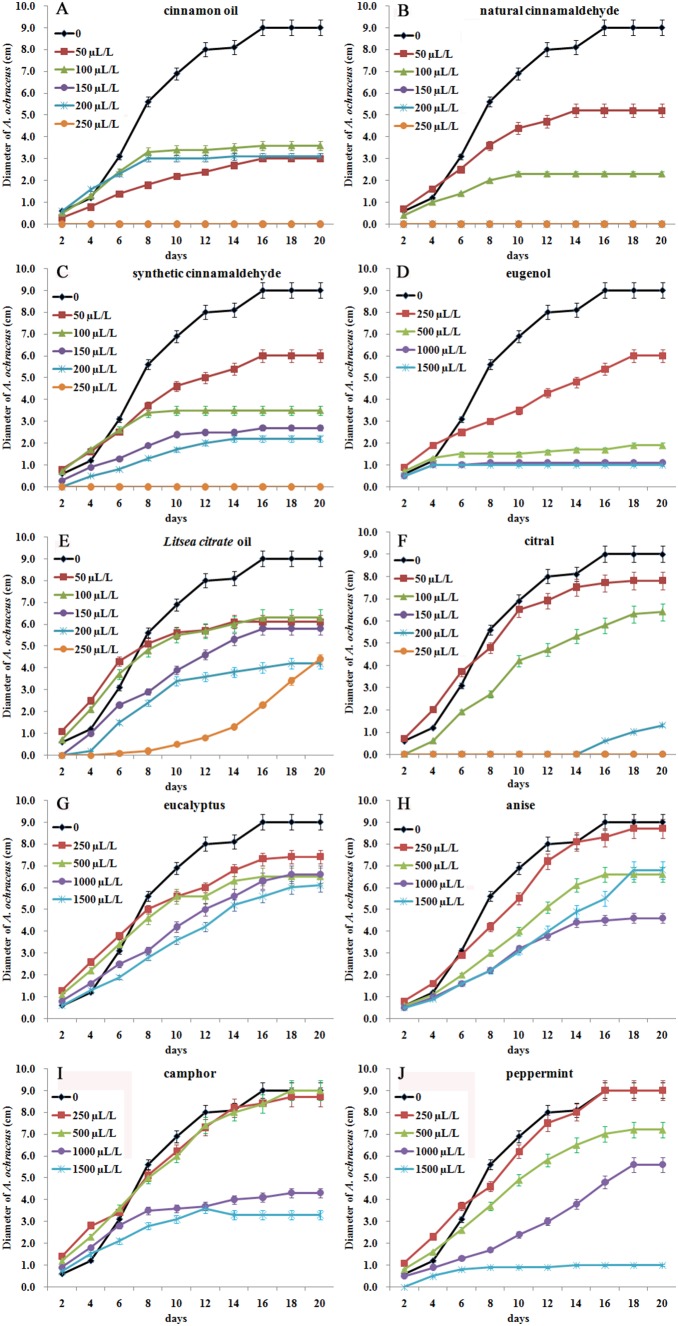
The effect of ten essential oils on the growth of *A. ochraceus* with fumigation.

Cinnamon oil, natural cinnamaldehyde, synthetic cinnamaldehyde, *L. citrate* oil and citral significantly inhibited the growth of *A. ochraceus* at 50−250 µL per liter MEA medium (*P*<0.01). Natural cinnamaldehyde proved to be the most effective against *A. ochraceus*, followed by cinnamon oil, synthetic cinnamaldehyde, citral, *L. citrate* oil, eugenol, peppermint, anise, eucalyptus and camphor oils (Fig. 1). The inhibitory effect increased proportionally with concentration and was also affected by treatment duration. *A. ochraceus* growth was completely inhibited with natural cinnamaldehyde (95%) at 150 µL/L, cinnamon oil (85% cinnamaldehyde) at 250 µL/L, synthetic cinnamaldehyde (99%) at 250 µL/L, citral (96%) at 250 µL/L. After 20 days incubation, the plates showing no growth were incubated for a further 7 days after removal of EOs, and no visible growth of *A. ochraceus* mycelia was observed. These results suggested a fungistatic effect of natural cinnamaldehyde, cinnamon oil, synthetic cinnamaldehyde and citral at lower concentrations and a fungicidal effect at higher concentrations.

Eugenol, peppermint, eucalyptus, anise and camphor oils did not completely inhibit *A. ochraceus* growth at 250–1500 µL/L. The diameters of *A. ochraceus* colonies treated with 1000 and 1500 µL/L eugenol did not increase from 1.0±0.06 cm from 4 to 20 days. Diameters of colonies treated with 1500 µL/L peppermint oil did not change from 1.0±0.07 cm from 14 to 20 days.

### Inhibitory effect of ten essential oils on *A. ochraceus* growth with contact assay

**Figure 2:**
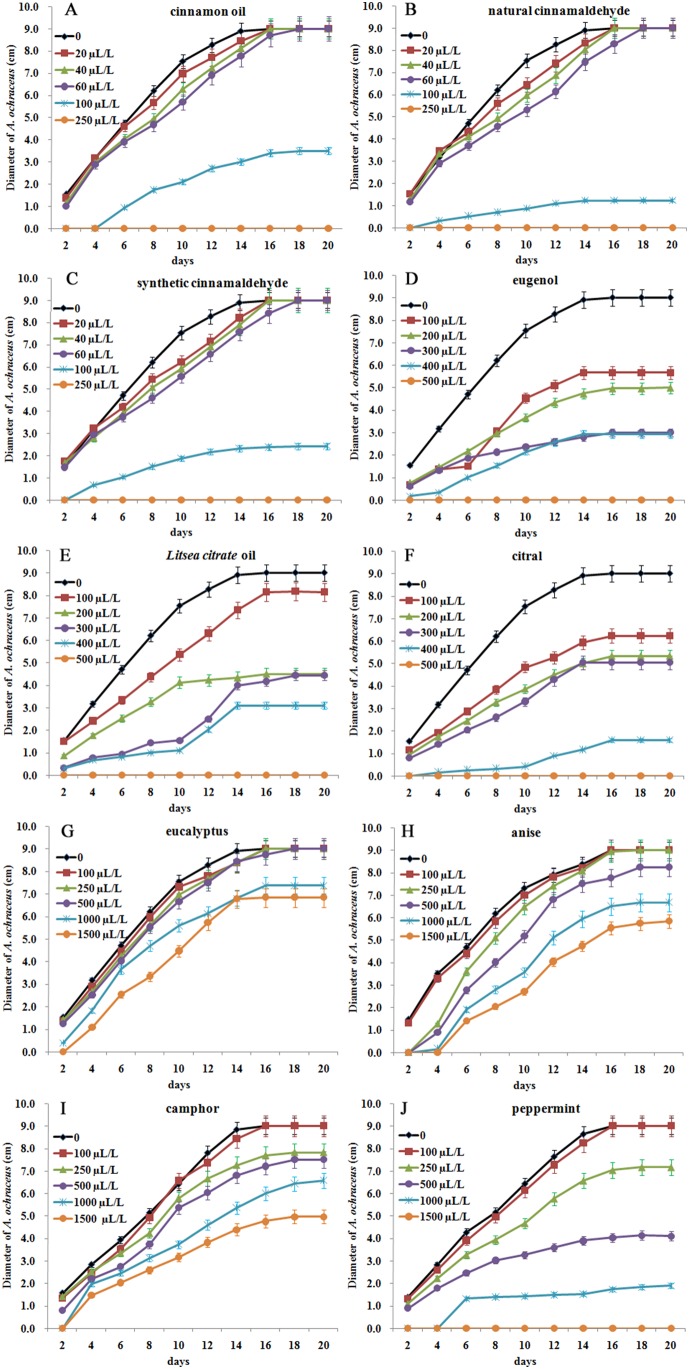
The effect of ten essential oils on the growth of *A. ochraceus* in contact assay.

Treatment with cinnamon oil, natural cinnamaldehyde, synthetic cinnamaldehyde, *L. citrate* oil, citral and eugenol significantly inhibited *A. ochraceus* growth at 100–500 µL/L on MEA medium (*P*<0.01). Treatment with peppermint oil significantly inhibited *A. ochraceus* growth at 500–1500 µL/L (*P*<0.01). However, eucalyptus, anise and camphor oils showed mild inhibition on *A. ochraceus* growth at 100–1500 µL/L. Natural cinnamaldehyde was highly effective against *A. ochraceus*, followed by synthetic cinnamaldehyde, cinnamon oil, citral, *L. citrate* oil, eugenol and peppermint oil (Fig. 2).

*A. ochraceus* growth was completely inhibited with natural cinnamaldehyde, synthetic cinnamaldehyde and cinnamon oil at 250 µL/L. For a complete antifungal effect, 500 µL/L of citral, *L. citrate* oil and eugenol were essential. At 1500 µL/L, peppermint oil completely inhibited the growth of *A. ochraceus*. However, eucalyptus, anise and camphor oils did not show complete inhibition at ≤1500 µL/L.

### MIC determined by contact assay

For *A. ochraceus*, the MIC of cinnamon oil (85%), natural cinnamaldehyde (95%) and synthetic cinnamaldehyde (99%) were 500 µL/L; those of *L. citrate* oil (85%) and citral (99%) were 800 and 700 µL/L, respectively (Table 1). Cells from the tubes of the five EOs at ≥500 µL/L were subcultured on MEA plates and no visible growth was observed, suggesting a permanent growth inhibition. The MIC of eugenol (99%) was 600 µL/L which was lower than the MIC of citral (99%), however inhibition by eugenol at 600–4000 µL/L was reversible. Eucalyptus, anise and camphor oils did not completely inhibit the growth of *A. ochraceus* at ≤40000 µL/L.

**Table 1:** MICs of the essential oils against *A. ochraceus* a.

Essential oils	MIC (µL/L)	Determine if the inhibitionwas reversible or permanent
Cinnamon oil (85% cinnamaldehyde)	500	Permanent (Without visible growth occurred)
Natural cinnamaldehyde (95%)	500	Permanent
Synthetic cinnamaldehyde (99%)	500	Permanent
Cinnamic alcohol (99%)	700	Permanent
*Litsea citrate* oil (85% citral)	800	permanent
Citral (99%)	700	Permanent
Eugenol (99%)	600	600–4000, reversible (visible growth occurred) ≥4000, permanent
Peppermint (50% methol)	2000	2000–40000, reversible ≥40000, permanent
Eucalyptus (80% cineole)	>40000	>40000, permanent
Anise (92% anethole)	>40000	Reversible
Camphor (55% bomeol)	>40000	Reversible

a Results are shown as minimum inhibitory concentrations (expressed as µL of essential oils added per L of liquid medium);

Reversible: visible growth occurred after subculturing on potato dextrose agar plates;

Permanent: without visible growth occurred after subculturing on potato dextrose agar plates.

### Scanning electron microscopy (SEM)

**Figure 3:**
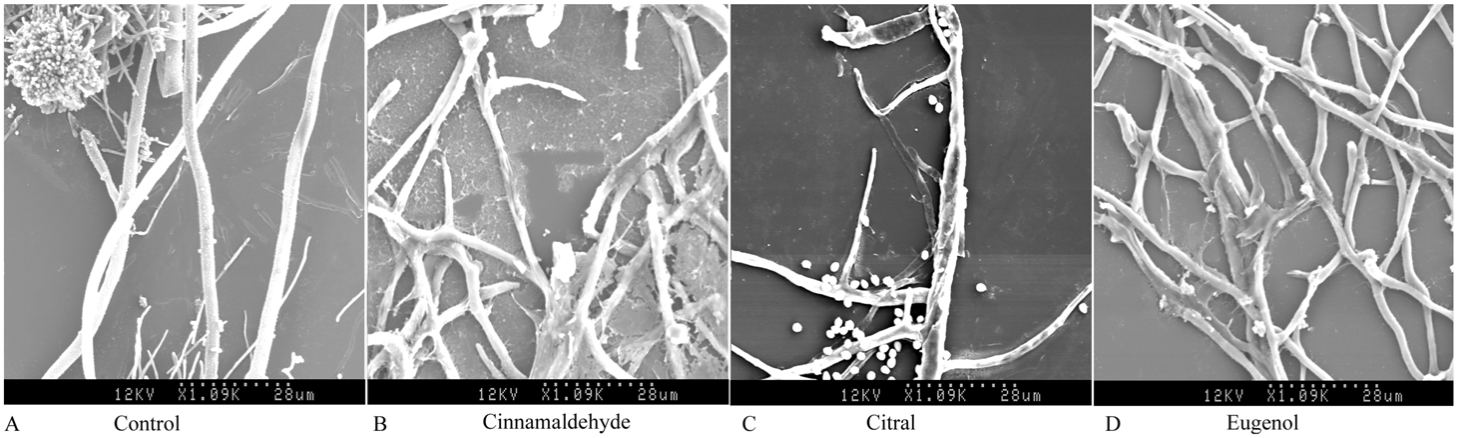
SEM for the nonfumigated and fumigated mycelia. A. Fumigated with 100 µL/L of natural cinnamaldehyde; B. Fumigated with 100 µL/L of citral; C. Fumigated with 250 µL/L of eugenol.

Morphological changes which occurred in the hyphae and conidiophores of *A. ochraceus* due to growing the fungus in MEA medium fumigated with 100 µL/L of natural cinnamaldehyde, 100 µL/L of citral and 250 µL/L of eugenol are shown in Fig. 3A–D. The growing nonfumigated healthy mycelia showed a normal morphology with linear, regular, homogenous and rough cell wall hyphae (Fig. 3A). The growing apexes of the hyphae were tapered with a smooth surface. However, this morphology underwent alterations following fumigation of the culture medium with natural cinnamaldehyde, citral and eugenol. The mycelia showed evident modifications in both apical regions and throughout the length of the hyphae. These hyphae were disrupted (Fig. 3B, D) and the apical regions of the haphae were modified and abnormal with rough surfaces (Fig. 3B, D). The hyphae became slender, shrank and winding and evident craters appeared on the cell wall (Fig. 3C). The conidiophores and vesicles were disrupted, and then the conidia were dispersed in disorder (Fig. 3C).

### Effect of three essential oils on ergosterol biosynthesis and OTA production

**Figure 4:**
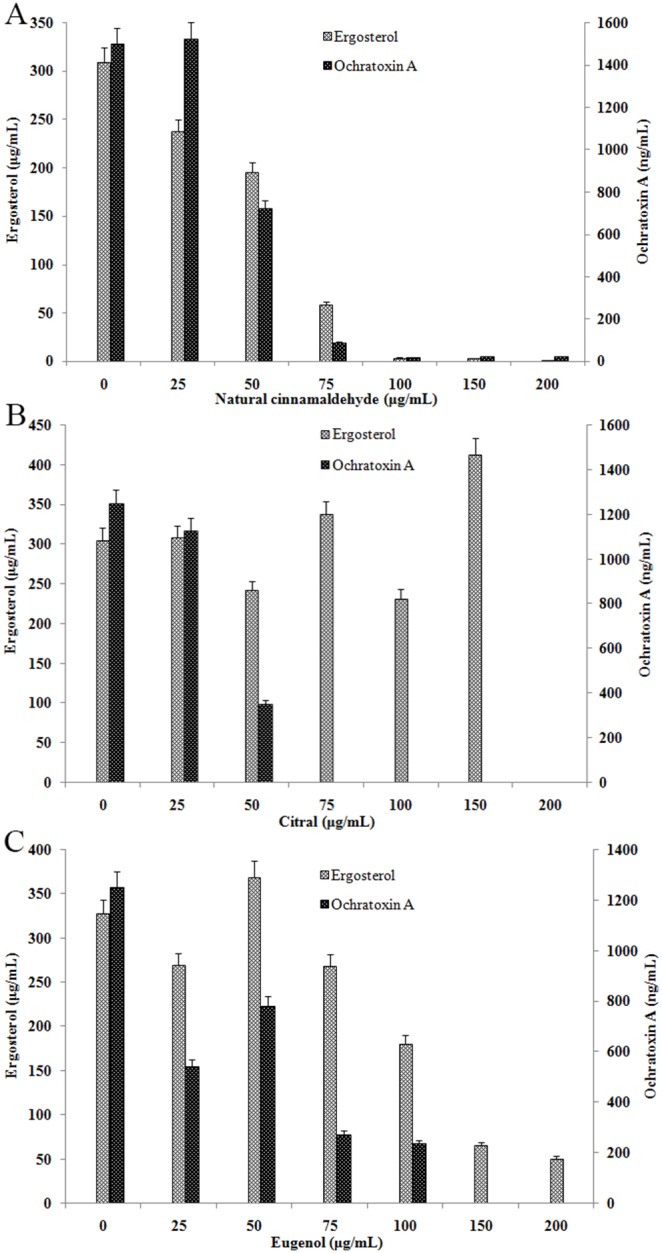
Effect of natural cinnamaldehyde (A), citral (B) and eugenol (C) on ergosterol biosynthesis and OTA production from *A. ochraceus* determined by HPLC. The experiment was conducted during 5 days of incubation under agitation at 28°C (n = 4).

Natural cinnamaldehyde at 25–50 µg/mL caused a mild reduction in ergosterol production and significantly inhibited ergosterol production in *A. ochraceus* at 75–200 µg/mL, with inhibition ranging from 80% to 100%. Natural cinnamaldehyde significantly inhibited production of OTA by *A. ochraceus* at lower concentrations (50–75 µg/mL) and almost completely inhibited production at higher concentrations (100–200 µg/mL) (Fig. 4A). Thus, cinnamaldehyde-induced ergosterol inhibition correlated with a reduction in OTA production. The decrease in OTA production was proportional to the decrease in fungal biomass. A similar result was found by Yamamoto-Ribeiro et al. (2013) [20]. Other studies demonstrated that EOs and compounds, limonene and thymol, inhibit fumonisin production by *F. verticillioides* [29], [30].

Citral at 25–150 µg/mL did not inhibit ergosterol biosynthesis and completely inhibited ergosterol production in *A. ochraceus* at 200 µg/mL (Fig. 4B). The significant oscillations were observed in ergosterol biosynthesis. Ergosterol biosynthesis was significantly induced by citral at 150 µg/mL. However, citral at 50 µg/mL significantly inhibited the OTA production and complete inhibition in OTA production was observed at 75–200 µg/mL.

Eugenol at 25–75 µg/mL caused oscillations in ergosterol production in *A. ochraceus* and effectively inhibited ergosterol production at 100–200 µg/mL, with inhibition ranging from 45% to 85% (Fig. 4C). This oscillation was reflected by an increase in ergosterol biosynthesis of *A. ochraceus* at 50 µg/mL eugenol compared with the control and significant inhibition at higher concentrations.

### Conversion of cinnamaldehyde by *A. ochraceus*

**Figure 5:**
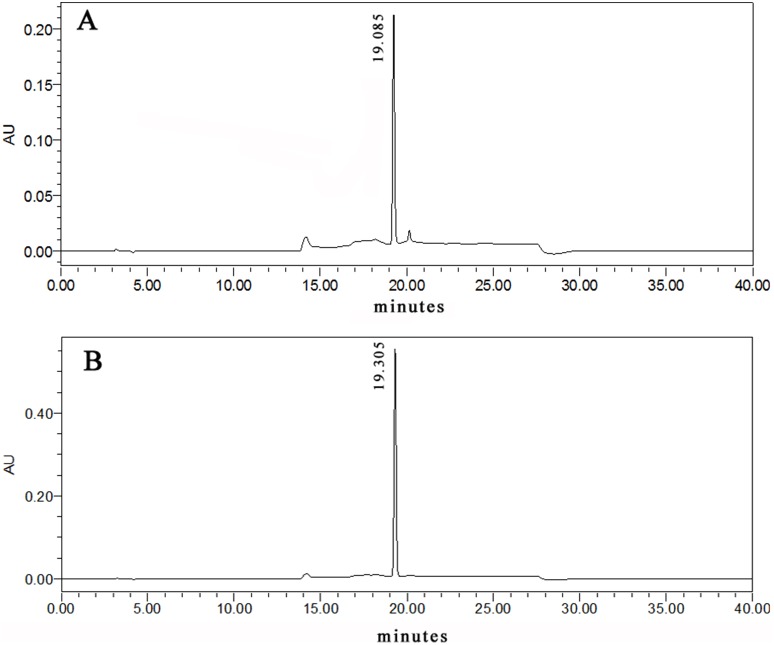
RP-HPLC detection of cinnamaldehyde of 50 mg/L (A) and cinnamic alcohol of 50 mg/L (B).

The changes in concentrations of cinnamaldehyde and its possible metabolite, cinnamic alcohol, in YES with *A. ochraceus* (treatment) and without fungal inoculation but with cinnamaldehyde (negative control) were monitored by RP-HPLC analysis (Fig. 5). The concentration of cinnamaldehyde before incubation at 28°C (0 h) was 64.56±4.26 mg/L with the negative controls and 64.83±5.33 mg/L with the treatment samples (Table 2). The result showed that only about 64.83% of the cinnamaldehyde added (100 mg/L) was extracted using ethyl acetate. After 2 h the concentration of cinnamaldehyde was stable in both the negative controls and treatment cultures. After 4 h the concentration of cinnamaldehyde decreased by 40 mg/L in treatment cultures, while it remained stable in the negative control during the tested time. However, with *A. ochraceus*, the concentration of cinnamaldehyde decreased to ≤4 mg/L at 8 h and was undetected at 12 h. The concentration of cinnamic alcohol was detected at 4 h and increased to about 55 mg/L at 8–12 h, then decreased to approximate 3 mg/L at 72 h and undetected at 96 h. However, the concentration of cinnamic alcohol was undetectable in the positive control during the tested time. Since cinnamic alcohol was detected at 4–72 h in treatment samples, its MIC was determined and found to be 700 mg/L and higher than that of cinnamaldehyde.

**Table 2:** Conversion of cinnamaldehyde to cinnamic alcohol by A. ochraceus* in YES.

Treatment	YES plus cinnamaldehyde, uninoculated (negative control)		YES plus *A. ochracesus* pluscinnamaldehyde, inoculated (treatment)		YES plus *A. ochraceus*(positive control)	
Concentration	Cinnamaldehyde(µg/mL)	Cinnamic alcohol(µg/mL)	Cinnamaldehyde(µg/mL)	Cinnamic alcohol(µg/mL)	Cinnamaldehyde(µg/mL)	Cinnamic alcohol(µg/mL)
0 h	64.56±4.26^a^	ND	64.83±5.33^a^	ND	ND	ND
2 h	64.13±3.21^a^	ND	62.54±4.26^a^	ND	ND	ND
4 h	63.41±4.65^a^	ND	24.17±1.30^b^	36.83±1.21^a^	ND	ND
8 h	62.84±5.11^a^	ND	3.41±0.34^c^	55.23±2.13^b^	ND	ND
12 h	62.25±4.32^a^	ND	ND	55.52±0.27^b^	ND	ND
24 h	62.14±4.21^a^	ND	ND	37.33±6.61^c^	ND	ND
48 h	61.71±2.26^ab^	ND	ND	14.82±2.48^d^	ND	ND
72 h	58.85±2.89^b^	ND	ND	3.12±0.39^e^	ND	ND
96 h	55.58±8.37^c^	ND	ND	ND	ND	ND

*ND, not detected. Different superscript lowercase letters in each column indicate a significant difference (*P*<0.05).

## Discussion

The 10 oils analyzed showed dose-dependent antifungal activity against *A. ochraceus* at the concentrations tested. This finding was consistent with the results obtained by Passone et al. (2012) [23] who reported that boldo (*Pëumus boldus* Mol.), poleo (*Lippia turbinate* var. *Integrifolia* [Griseb.]), clove (*Syzygium aromaticum* L.), anise (*Pimpinella anisum*) and thyme (*Thymus vulgaris*) EOs were effective non-toxic biopreservatives against *Aspergillus* section *Nigri* growth and OTA contamination *in* *vitro*. Boldo EO showed the greatest effect on fungal isolates with significant reductions at 1000 and 2000 µL/L and total growth inhibition at 3000 µL/L. *Matricaria chamomilla* L. oil was a potent inhibitor of *A. niger* growth *in* *vitro* with a growth inhibition at oil concentration of 15.62 µg/mL (∼7.5%) and reached a maximum of ∼92.5% at the final concentration of 1000 µg/mL [31].

Although the concentrations of oils tested in this study were not the same, the headspace volatile assays showed greater inhibition than the contact assays with the exception of eugenol and peppermint oil. Complete growth inhibition of *A. ochraceus* was verified at 150–250 µL/L by headspace volatile assays and 250–500 µL/L in the contact assays for cinnamon oil, natural cinnamaldehyde, synthetic cinnamaldehyde, *L. citrate* oil and citral. In contrast, Passone et al. (2012) [23] studies showed that boldo, poleo and clove essential oils have higher inhibition effect in contact assays than headspace volatile assays. Complete growth inhibition was observed at ≥1500 µL/L for boldo, poleo and clove oils in contact assays and ≥2000 µL/L for boldo in headspace volatile assays, while full fungal growth was not observed with 3000 µL/L of poleo and 5000 µL/L of clove oils. These results showed that both assays are necessary to evaluate the inhibitory effects of essential oils. Ergosterol has hormonal and structural effects in fungal cells and is responsible for regulating the flow and activity of membrane-bound enzymes [32]. Some antifungal agents inhibit fungal growth by interrupting ergosterol biosynthesis through binding to ergosterol in membranes. This damages the integrity and function of membrane-bound proteins and disturbs osmosis, fungal growth and proliferation [32]. In our study, natural cinnamaldehyde at 25–50 µg/mL caused a mild reduction in ergosterol production and significantly inhibited ergosterol production in *A. ochraceus* at 75–200 µg/mL, with inhibition ranging from 80% to 100%. Various research groups have reported the oscillations in ergosterol biosynthesis [20], [28], [33]. Yamamoto-Ribeiro et al. (2013) [20] found that *Zingiber officinale* essential oil increases ergosterol biosynthesis of *F. vertcillioides* at 100 µg/mL compared with the control group and reduces or completely inhibits ergosterol biosynthesis at higher concentrations. Lucini et al. (2006) [33] found that monoterpenes at low concentrations lead to lipid peroxidation in fungi, inducing an adaptive response and reprogramming genomic expression to protect the cell wall structure, and consequently increase ergosterol biosynthesis. Dabolena et al. (2008) [29] found that limonene, menthol, menthone, and thymol at 75 µg/mL could increase ergosterol production by *F. verticillioides*.

In general, natural cinnamaldehyde and eugenol slightly inhibited biosynthesis of ergosterol and OTA at lower concentrations and significantly or completely inhibited biosynthesis at higher concentrations. The reductions of OTA production caused by cinnamaldehyde and eugenol at 25–100 µg/mL were positively correlated with EOs-induced ergosterol inhibition. Therefore, the decrease in OTA production was proportional to the decrease in fungal biomass at some levels. This result was similar with the findings of Yamamoto-Ribeiro et al. (2013) [20] and Dambolena et al. (2010) [34]. Their studies reported that essential oils from *Zingiber officinale*, *Ocimum basilicum* L. and *Ocimum gratissimum* L. inhibited fumonisin production by *F. verticillioides* and the inhibition was proportional to the decrease in fungal biomass.

However, citral showed a different effect than natural cinnamaldehyde and eugenol. Citral at 75–150 µg/mL completely inhibited OTA production but not ergosterol biosynthesis. This result suggested that the decrease in OTA production induced by citral was not due to the decrease in fungal biomass but might be due to suppression of transcription of OTA biosynthesis genes. The polyketide synthase gene (*pks* gene) is responsible for the synthesis of the polyketide dihydroisocoumarin and involved in the first steps of the OTA biosynthetic pathway. Real-time quantify PCR (qPCR) analysis confirmed that the transcription of *pks* gene was completely inhibited by 75–150 µg/mL of citral. A similar inhibition of OTA production was observed with higher concentration of eugenol. At lower concentrations (25–100 µg/mL), the decrease in OTA production by eugenol was proportional to the decrease in fungal biomass. However, at 150–200 µg/mL OTA production was completely inhibited by eugenol but ergosterol production was not fully inhibited. This result suggested that inhibition of OTA required a lower oil concentration of EO than the inhibition of fungal growth. This finding agrees with results from Passone et al. (2012) [23], who reported complete inhibition of OTA production by *Aspergillus niger* RCP42 at ≥1000 µL/L of clove oil, whose main component is eugenol, while total fungal growth inhibition did not occur at 3000 µL/L at *a*_w_ 0.98. Complete inhibition of OTA production by *Aspergillus carbonarius* RCP203 occurred at ≥1000 µL/L of clove oil, while total fungal growth inhibition did not occur at 3000 µL/L at *a*_w_ 0.93. Murthy et al. (2009) [35] found that fungal growth and OTA production decreased progressively with increases in Ajowan ethanolic extract (AEE) concentration. At 250 µL/mL, they could see fungal growth and OTA production being inhibited. However, the inhibition towards fungal growth and OTA biosynthesis was not linear. At 50 and 150 µL/g of AEE, inhibition of OTA production was higher than inhibition of fungal growth. Jayashree and Subramanyam (1999) [36] found that eugenol up to 0.75 mmol/L inhibited aflatoxin production with no significant effect on *Aspergillus parasiticus* growth. Furthermore, they found that eugenol inhibited the lipid peroxidation in aflatoxin production with no significant inhibition of growth or primary metabolism. However, an inverse correlation was shown by Pereira et al. (2006) [37] who observed that clove oil could completely inhibit the mycelial growth of *A. ochraceus*, but no significant affect in antiochratoxigenic activity.

This is the first study to show that *A. ochraceus* can convert cinnamaldehyde to cinnamic alcohol at ≤12 h, and cinnamic alcohol was further degraded and no new metabolites were detected by RP-HPLC. The degradation might be due to *Aspergillus* genus has several enzymes such as alcohol dehydrogenase and aldehyde reductase that catalyze cinnamaldehyde to cinnamic alcohol [38], [39]. Similar result was obtained by Vsivalingam et al. (2013) [40], who firstly found that *E. coli* O157:H7 could convert cinnamaldehyde to cinnamic alcohol by alcohol dehydrogenase and 2,5-diketo-D-gluconate reductase A.

## Conclusions

The inhibitory effect of 10 essential oils on *A. ochraceus* growth was tested with fumigation and contact assays. Natural cinnamaldehyde proved to be the most effective against *A. ochraceus*, followed by cinnamon oil, synthetic cinnamaldehyde, citral, *L. citrate* oil, eugenol and peppermint oil. Headspace volatile assays showed higher inhibition than contact assays with exception of eugenol and peppermint oil. SEM of *A. ochraceus* cells fumigated with natural cinnamaldehyde, citral and eugenol showed alteration in the morphology of the hyphae which appeared collapsed, and abnormal branching of hyphae in apical region and loss of linearity. The inhibitory effects of natural cinnamaldehyde, citral and eugenol on ergosterol biosynthesis and OTA production indicated that natural cinnamaldehyde had a higher antifungal activity than citral and eugenol. However, natural cinnamaldehyde had a lower antiochratoxigenic effects than citral and eugenol. These results suggested that the inhibition of OTA production by natural cinnamaldehyde was mainly due to a decrease in fungal biomass. However, in addition to antifungal activity, citral and eugenol could significantly inhibit the OTA biosynthesis pathway of *A. ochraceus*.
